# Ultrathin high-*κ* antimony oxide single crystals

**DOI:** 10.1038/s41467-020-16364-9

**Published:** 2020-05-19

**Authors:** Kena Yang, Tao Zhang, Bin Wei, Yijia Bai, Shuangfeng Jia, Guanghui Cao, Renhui Jiang, Chunbo Zhang, Enlai Gao, Xuejiao Chang, Juntao Li, Simo Li, Daming Zhu, Renzhong Tai, Hua Zhou, Jianbo Wang, Mengqi Zeng, Zhongchang Wang, Lei Fu

**Affiliations:** 1 College of Chemistry and Molecular Sciences, Wuhan University, Wuhan, 430072 China; 2 Department of Quantum Materials Science and Technology, International Iberian Nanotechnology Laboratory (INL), Avenida Mestre Jose Veiga, Braga, 4715-330 Portugal; 3 College of Chemical Engineering, Inner Mongolia University of Technology, Hohhot, 010051 China; 4 School of Physics and Technology, Center for Electron Microscopy, MOE Key Laboratory of Artificial Micro- and Nano-structures, and Institute for Advanced Studies, Wuhan University, Wuhan, 430072 China; 5 Department of Engineering Mechanics, School of Civil Engineering, Wuhan University, Wuhan, 430072 China; 6 Shanghai Synchrotron Radiation Facility, Shanghai Institute of Applied Physics, Chinese Academy of Sciences, Shanghai, 201204 China; 7 X-ray Science Division, Advanced Photon Source, Argonne National Laboratory, Argonne, IL 60439 USA

**Keywords:** Two-dimensional materials

## Abstract

Ultrathin oxides have been reported to possess excellent properties in electronic, magnetic, optical, and catalytic fields. However, the current and primary approaches toward the preparation of ultrathin oxides are only applicable to amorphous or polycrystalline oxide nanosheets or films. Here, we successfully synthesize high-quality ultrathin antimony oxide single crystals via a substrate-buffer-controlled chemical vapor deposition strategy. The as-obtained ultrathin antimony oxide single crystals exhibit high dielectric constant (~100) and large breakdown voltage (~5.7 GV m^−1^). Such a strategy can also be utilized to fabricate other ultrathin oxides, opening up an avenue in broadening the applicaitons of ultrathin oxides in many emerging fields.

## Abstract (web summary)

The ultrathin oxide nanosheets obtained through previous approaches usually exhibit amorphism or polycrystallinity, which limit their properties towards electronic devices. Here, the authors synthesize ultrathin antimony oxide single crystals with high dielectric constant (~100) and large breakdown voltage (~5.7 GV m^−1^).

## Introduction

Ultrathin oxides have been reported to possess excellent properties in electronic, magnetic, optical, and catalytic fields^1–6^. However, the ways to simply synthesize ultrathin oxides through a general and facile method is a big challenge. The current and primary approaches toward the preparation of ultrathin oxides arise from wet-chemistry synthesis, where a variety of ultrathin metal oxides can be attained^7–9^. In addition, the liquid metal-based reaction route, which requires metals that can be co-alloyed into the melt, has been utilized to obtain low-dimensional metal oxides^1^. However, these methods are only applicable to amorphous or polycrystalline oxide nanosheets or films with degraded properties^1,7–9^. Recently, Li et al. presented an approach for the preparation of ultrathin high-*κ* oxide, which showed the potential for two-dimensional electronic devices^10^. However, it is prerequisite to grow a molecular crystal as a seeding layer for the uniform deposition of the ultrathin oxide. Thus far, the research for high-quality and reproducible ultrathin high-*κ* oxide single crystals has not been achieved yet, albeit this is highly desirable.

In this work, we realize the growth of ultrathin antimony oxide single crystals (down to 1.8 nm) based on a specially substrate-buffer-controlled chemical vapor deposition (SBC–CVD) strategy. After the growth, synchrotron-based X-ray diffraction (XRD) and high-resolution transmission electron microscopy (HRTEM) are conducted to elucidate the structure of such ultrathin antimony oxide single crystals. The SbO_1.93_ single crystals exhibit excellent insulating capabilities with high dielectric constant (~100) and large breakdown electric field (~5.7 GV m^−1^). Such ultrathin antimony oxide single crystals will facilitate the ongoing research of the applications of ultrathin oxides.

## Results

### The growth and characterizations of ultrathin antimony oxide single crystals

**Fig. 1:**
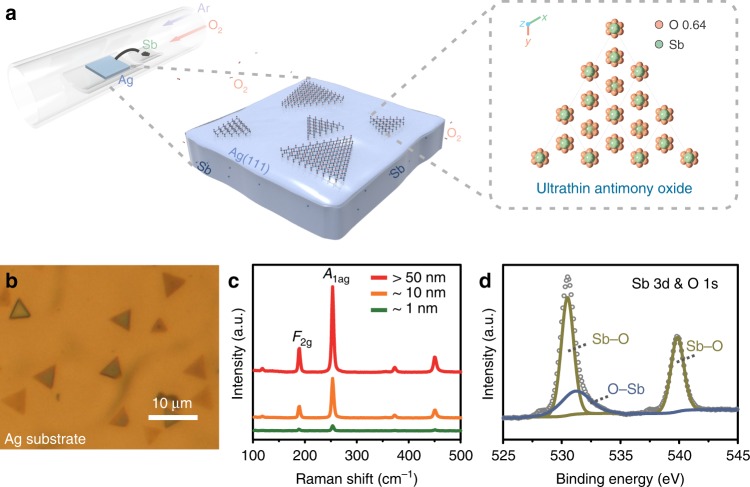
Growth and spectral characterizations of ultrathin antimony oxide crystals. **a** Schematic of the strategy for the growth of ultrathin antimony oxide. **b** OM image of ultrathin antimony oxide crystals grown on the re-solidified Ag substrate. **c** Raman spectra of ultrathin antimony oxide crystals with different thicknesses. **d** XPS spectra of the ultrathin antimony oxide.

The strategy for the growth of ultrathin antimony oxide single crystals is schematically illustrated in Fig. 1a. Before the growth, a re-solidified Ag(111) substrate is prepared by a designed high-temperature (1000 °C) annealing process (details in Supplementary Figs. 1–6). Then, to achieve the growth of ultrathin antimony oxide single crystals, commercial antimony powder is placed upstream to provide antimony vapor, and the Ag substrates are placed in the downstream area with temperature T = 750 °C. The growth starts while O_2_ is introduced into the system, and the growth process is maintained for a few minutes. Typical optical microscope (OM) image of the ultrathin antimony oxide is shown in Fig. 1b, which reveals that these triangular crystals exhibit a uniform size of about 5 μm, in agreement with the scanning electron microscope (SEM) data (Supplementary Fig. 7a). Raman spectra of the as-grown triangular crystals are shown in Fig. 1c, revealing that antimony oxide with varying thickness corresponds to the homologous peaks. The only difference is that the Raman spectra intensity shows a linear relation with the thickness. As shown in Supplementary Fig. 7b, Raman intensity maps using the *A*_1g_ mode demonstrate a uniform response over the entire crystal, which proves the uniformity of the ultrathin antimony oxide crystal. Then, X-ray photoelectron spectroscopy (XPS) is implemented to further characterize the chemical composition of the as-prepared ultrathin antimony oxide crystals (Fig. 1d). The peaks at 530.3 and 539.6 eV are attributed to the Sb 3d_5/2_ and 3d_3/2_ states, and the peak at 531.3 eV is corresponding to the O 1 s state, respectively^11^. Due to that the reaction Gibbs free energy of Sb with O_2_ (−110.97 kJ mol^−1^) is much lower than that of Ag (−7.84 kJ mol^−1^)^12^, Sb will react with oxygen first. But the amount of O_2_ must be limited to avoid the oxidation of the Ag substrate. To achieve the complete oxidation of Sb to form ultrathin antimony oxide single crystals, the amount of elementary Sb should also be controlled. Here, the Ag substrate serves as a buffer to achieve a modest supply of Sb. Such SBC–CVD mechanism is confirmed by the XPS depth profile. From the valence analysis of Sb along a surface normal-direction (Supplementary Fig. 8), it can be seen that, with the removal of the surface layer, the Sb^0^ appears and keeps at a constant amount within the whole Ag substrate, which demonstrates that the excess Sb is buffered within the substrate, and it is very critical for the complete oxidation of Sb and the subsequent growth of ultrathin antimony oxide crystals.

### Structure elucidation of ultrathin antimony oxide

**Fig. 2:**
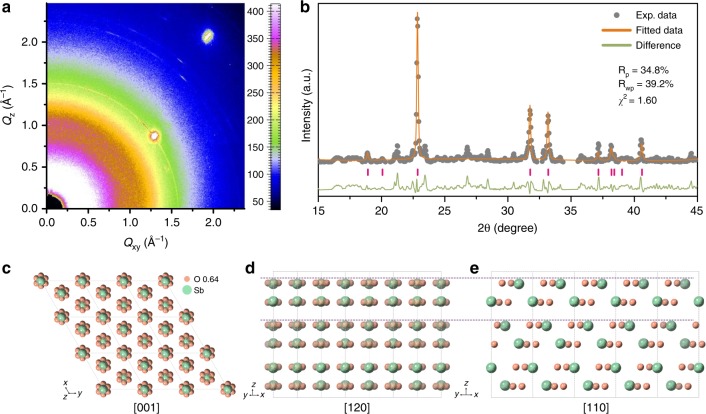
Structure elucidation of ultrathin antimony oxide. **a** Synchrotron-based GI-XRD image of ultrathin antimony oxide. **b** The fitting of GI-XRD data. **c**–**e** Schematic of the fitted SbO_1.93_ structure along the [001] (**c**), [120] (**d**), and [110] (**e**) directions.

To further reveal the structure of ultrathin antimony oxide, synchrotron-based grazing-incidence XRD (GI-XRD) is conducted. As shown in Fig. 2a, the diffraction patterns are recorded on the image plate for the ultrathin antimony oxide. Then, owing to the similarity between the GI-XRD pattern of ultrathin antimony oxide and the simulated XRD pattern of β-antimony (Supplementary Fig. 9), the structure of β-antimony is used as the initial model for the structure creation of ultrathin antimony oxide. The rough crystal cell parameters can be obtained, and the preliminary cell structure can be parsed out. Then, the preliminary cell structure is further refined by Generalized Structure and Analysis Software (GSAS) package^13^ (Supplementary Note 1). Figure 2b shows the experimental and the fitted data, where there is only a slight difference between them. From the diffraction result, the structure of the ultrathin antimony oxide can be elucidated, which is shown in Fig. 2c. The atomic ratio of Sb to O is 1:1.93, then the chemical formula can be defined as SbO_1.93_. And the top view and side view are demonstrated in Fig. 2d and e, respectively. Specifically, the crystal data and structure refinement for SbO_1.93_ are listed in Supplementary Table 1. Atomic coordinates, occupancy factors (*f*_occ_) and isotropic displacement parameters (*U*_iso_) are listed in Supplementary Table 2. Bond lengths and bond angles are listed in Supplementary Table 3. The unit-cell parameters and lattice plane parameters are listed in Supplementary Table 4. To verify the rationality of the final structure, we compared the as-obtained XRD pattern of the SbO_1.93_ sample with the simulated XRD patterns of β-antimony and theoretical SbO_1.93_ structure. As shown in Supplementary Fig. 9, the XRD pattern of SbO_1.93_ sample is well consistent with the simulated XRD pattern of theoretical SbO_1.93_ structure. Owing to oxygen-containing structure, both the peak position and the peak intensity of SbO_1.93_’s XRD pattern have changed. The comparison between the structure of β-antimony and that of SbO_1.93_ is exhibited in Supplementary Fig. 10, from which the similar atomic configuration can be observed in both vertical and side views. Moreover, as shown in Supplementary Fig. 10, it can be found that both β-antimony and SbO_1.93_ present a hexagonal symmetry with R${\bar{3}}$m space group (no. 166).

**Fig. 3:**
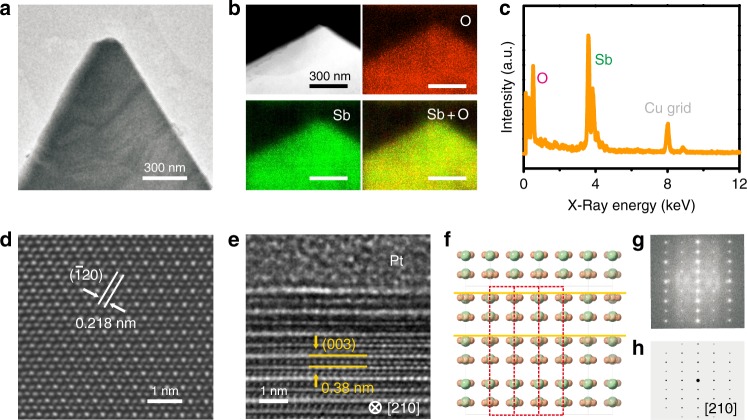
Atomic-scale analysis of the ultrathin SbO_1.93_ crystal. **a** TEM image of an ultrathin SbO_1.93_ crystal. **b** STEM image and corresponding EDS elemental mapping of O, Sb, and their overlap for the SbO_1.93_ crystal. **c** The TEM-EDS spectrum of the SbO_1.93_ crystal. **d** HRTEM image of the R-3m SbO_1.93_ crystal. **e** Cross-sectional TEM image of the SbO_1.93_ lamella along the [210] direction. **f** Schematic of the fitted SbO_1.93_ structure viewing from [210] direction. **g** FFT from the area in **e**. **h** The simulated SAED pattern of SbO_1.93_ along the [210] zone axis.

We further use TEM and energy dispersive X-ray spectroscopy (EDS) to confirm the composition and the crystal structure of the ultrathin SbO_1.93_. Figure 3a shows the TEM image of an ultrathin SbO_1.93_ crystal that is transferred onto the copper grid through water-soluble polyvinyl alcohol (PVA) mediator^14^. As displayed in Fig. 3b, the EDS mapping reveals the uniform spatial distribution of Sb and O in the crystal. Meanwhile, the EDS spectrum also confirms the existence of the element of Sb and O in the crystal (Fig. 3c). Figure 3d shows a typical high-resolution TEM (HRTEM) image, which clearly resolves a $\bar{1}20$ lattice (2.18 Å) with a periodic atomic distribution. This result is in a good agreement with the fitted lattice in Supplementary Table 4 and it further support the ${\mathrm{R}}{\bar{3}}{\mathrm{m}}$ symmetry of the ultrathin SbO_1.93_, which was totally different from the structures of other common antimony oxide (Supplementary Fig. 11 and Supplementary Note 2). Besides, we have conducted cross-sectional TEM characterizations to confirm the arrangement in the direction perpendicular to the Sb–O triangular lattice plane. In detail, the ultrathin SbO_1.93_ crystal was first protected by a Pt layer, and then the cross-section TEM sample was prepared by focused ion beam (FIB). After that, cross-sectional TEM characterization was conducted, and corresponding results are displayed in Fig. 3e–h. Figure 3e shows the cross-sectional TEM image of the ultrathin SbO_1.93_ lamella along the [210] direction, with a clearly resolved interlayer distance of 0.38 nm, which corresponds to the distance of the (003) lattice. From the schematic of the fitted SbO_1.93_ structure viewing from [210] direction (as shown in Fig. 3f), it can be concluded that the interlayer distance is one third than the distance of the c-axis lattice, which is in good agreement with the cross-sectional TEM results. Besides, the fast Fourier transform (FFT) pattern exhibited in Fig. 3g is well consistent with the simulated selected area electron diffraction (SAED) pattern along the [210] zone axis (Fig. 3h), which further confirms the as-proposed arrangement in the perpendicular direction. Furthermore, Supplementary Fig. 12 shows the experimental SAED images along [001], [110], and [100] directions, which match well with the simulated SAED patterns, confirming that the as-simulated structure is correct and reasonable.

### The insulating property of the ultrathin SbO_1.93_

**Fig. 4:**
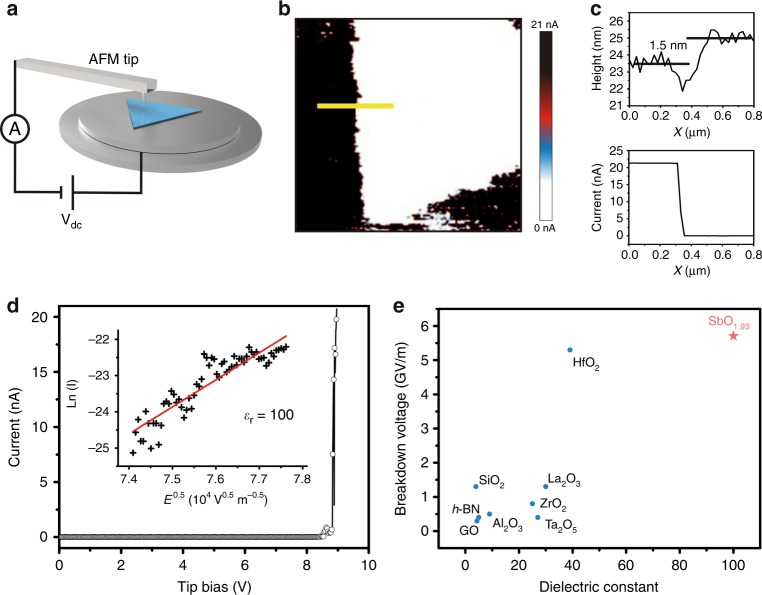
Conductive AFM test for the ultrathin SbO_1.93_. **a** Schematic of the peak force tunneling AFM (PF-TUNA) setup. **b** AFM current maps for the edge region of an ultrathin SbO_1.93_ crystal on Ag substrate. **c** The height (top) and current (bottom) profiles correspond to the region in **b** indicated by the yellow line. The Ag region and the SbO_1.93_ region are corresponding to the left and right sides, respectively. **d** Current–voltage curve measured through the ultrathin SbO_1.93_ crystal. Inset: fit to the Schottky emission model and the determined dielectric constant. **e** Dielectric constant and breakdown voltage of typical high-*κ* materials, graphene oxide, *h*-BN, and SbO_1.93_ in this work.

To further characterize the insulating property of the ultrathin SbO_1.93_ crystals, we conduct atomic force microscopy (AFM) investigation of the as-prepared ultrathin SbO_1.93_ crystals on the Ag substrate. The ultrathin SbO_1.93_ crystals show a smooth appearance under the AFM imaging. The different thickness (1.8 nm, 6 nm, 8 nm, and 16 nm) of the ultrathin SbO_1.93_ crystals can be seen in Supplementary Fig. 13. After that, conductive AFM characterizations are implemented to investigate the insulating property of the as-grown ultrathin SbO_1.93_ crystals. Due to that silver is a good conductor, the as-prepared ultrathin SbO_1.93_ crystals on the Ag substrate is directly used for the conductive AFM test (Fig. 4a). As shown in Fig. 4b, the tested ultrathin SbO_1.93_ crystal is completely insulating and pinhole-free, despite that it is only ~1.5 nm thick (Fig. 4c). The breakdown electric field, defined as the field at which the current rises above the noise level, is measured to be ~5.7 GV m^−1^, as determined by the current–voltage (I–V) characterization of the ultrathin SbO_1.93_ crystal (Fig. 4d). This value is one order of magnitude higher than the breakthrough field for CVD-grown multilayer *h*-BN^15^, highlighting the excellent quality of the ultrathin SbO_1.93_ crystals. Fitting the I–V curve to the Schottky emission model allows us to determine a dielectric constant of ~100 for the ultrathin SbO_1.93_ crystals (inset of Fig. 4d; Supplementary Note 3)^16,17^. Furthermore, we analyze the low-loss region of the electron energy loss spectrum (EELS) of the ultrathin SbO_1.93_ crystal and found that the band gap of the ultrathin SbO_1.93_ is ~6.3 eV (Supplementary Fig. 14; Supplementary Note 4). It is noteworthy that, compared with other oxides (such as SiO_2_, Al_2_O_3_, ZrO_2_, Ta_2_O_5_, La_2_O_3_, HfO_2_, and graphite oxide (GO))^18–20^ and *h*-BN^15^, SbO_1.93_ has a better electrical isolation property (Fig. 4e), which holds substantial promise for future miniaturized electronic and optoelectronic devices.

To probe whether such ultrathin antimony oxide could act as a compatible oxide layer for antimonene, a hydrogen-reduction process is conducted. The reduction process is shown schematically in Supplementary Fig. 15a. The Raman spectrum of antimonene/antimony oxide is different from that of the pure ultrathin antimony oxide, and the peak at 152 cm^−1^ corresponds to the *A*_1g_ of antimonene, confirming that antimonene has been obtained after reduction (Supplementary Fig. 15b)^21^. The results are further confirmed by XPS. As shown in Supplementary Fig. 15c, peaks at 528.0 and 537.3 eV are attributed to Sb^0^. In comparison with the result of pure antimony oxide crystals, the relative intensity of these two peaks increase significantly, which proved the existence of antimonene. Supplementary Fig. 15d shows the photoluminescence (PL) properties of the antimonene/antimony oxide under 355-nm laser excitation, and the emission peak of antimonene is located at 560 nm (2.21 eV), which is consistent with the theoretical prediction of the monolayer antimonene^21^. Time-resolved photoluminescence (TRPL) decays monitored at 560-nm emission peak (excitation wavelength 400 nm) are collected to provide further insights. The PL decays shown in Supplementary Fig. 15e can be fitted by a single exponential function, and τ is 2.8 ns. This suggests the existence of one pathway for the electron–hole radiative recombination from one emission species, which is corresponding to the emission of monolayer antimonene. The electronic property of antimonene/antimony oxide is also investigated. The electrical contact is defined by standard electron beam lithography followed by metal deposition of 10-nm-thick chromium and 30-nm-thick gold. The electrical characterization of the fabricated antimonene/antimony oxide devices is performed using a semiconductor parameter analyzer at room temperature. Schematic illustration of the device structure of antimonene/antimony oxide is shown in Supplementary Fig. 15f. Supplementary Fig. 15g shows the current–voltage (I_ds_–V_ds_) characteristics of antimonene/antimony oxide. Different from the electrical isolation properties of ultrathin antimony oxide (Supplementary Fig. 16), the antimonene/antimony oxide shows semiconducting feature, like the intrinsic antimonene.

## Discussion

In conclusion, we report the growth of high-quality ultrathin antimony oxide single crystals based on the SBC–CVD strategy. To elucidate the structure of ultrathin antimony oxide, synchrotron-based XRD and HRTEM are conducted. This type of high-quality ultrathin insulators has a high dielectric constant (~100) and a large breakdown electric field (~5.7 GV m^−1^). Furthermore, such strategy is not limited to antimony oxide, allowing to fabricate other oxide, such as bismuth oxide, germanium oxide and tin oxide (details in Supplementary Figs. 17–19 and Supplementary Table 5). We envision that this strategy can be expanded to construct more other ultrathin oxide crystals, thereby advancing the practical application of ultrathin oxides.

## Methods

### CVD growth of ultrathin antimony oxide crystals

To achieve the growth of ultrathin antimony oxide crystals, we utilized a re-solidified Ag substrate. At first, a piece of Ag wire was placed on Co foil. Under the protection of an Ar/H_2_ atmosphere, the Ag spread evenly over the entire foil by annealing at 1000°C for ∼20 min. After that, the as-obtained Ag(111) substrate was utilized for the CVD growth reaction. During the synthesis process, the antimony powder was placed in the upstream, and the furnace was heated up to 750 °C. The vapor of Sb was carried downstream by 20 sccm Ar, 5 sccm H_2_ and O_2_. And then Sb atoms react with O_2_ to grow ultrathin antimony oxide on the substrates. After the growth process, the furnace was cooled down fast to room temperature.

### The transfer of ultrathin antimony oxide to the copper grid and SiO_2_/Si substrate

At first, the 9 wt% PVA (Alfa Aesar, 98–99% hydrolyzed, high-molecular-weight) aqueous solution was spin-coated on the top of a smooth base. The pre-spin speed is 1000 rpm (time = 10 s) and then the spin speed is 3000 rpm (time = 60 s), followed by baking at 100 °C for 60 s on a heating stage. After the baking process, the polymer mediator has been obtained. Then the PVA film was covered on the sample and heated to 80 °C for half an hour. After that, the PVA film was transferred from the growth substrate to the target substrate. The polymer mediator was removed by dissolving in deionized water at 50 °C for ∼2 h, and finally, the ultrathin antimony oxide single crystals were left on the target substrate.

### Characterization

Optical images were taken with an optical microscope (Olympus DX51). Raman spectra with an excitation wavelength of 532 nm were carried out using a Renishaw inVia, and Raman mapping was taken by WITec alpha 300 R with an excitation wavelength of 488 nm. SEM images were obtained from a ZEISS Merlin Compact SEM with EDS spectra collected by X-MaxN Oxford EDS. X-ray photoelectron spectroscopy was performed on a Thermo Scientific, ESCALAB 250Xi. The binding energies were calibrated by referencing the C 1 s peak (284.8 eV). The thickness was measured on a NT-MDT Ntegra Spectra atomic force microscope. The conductive AFM characterizations were also conducted on NT-MDT Ntegra Spectra. TEM images were taken by FEI Titan themis 200 operated at an accelerating voltage of 80 kV. The antimony oxide lamellas for the cross-sectional TEM characterization were prepared via FIB treating by FEI Helios NanoLab 450 S DualBeam-FIB with UHREM FEG-SEM. The information of element distribution was also collected with EDS (INCAPentalFETx3 Oxford). The GI-XRD of the ultrathin antimony oxide were recorded at the BL14B1 beamline of Shanghai Synchrotron Radiation Facility (SSRF), and the data were calibrated with the LaB_6_ standard sample. The incident photon energy was 10 keV (wavelength = 1.2398 Å) at an incident angle of 0.5°, and the diffracted X-rays were collected at an interval of 80 s using the 2D Mar225 CCD detector. XRD measurements for the re-solidified Ag substrates were performed using a Rigaku MiniFlex600 with Cu–K*α* radiation over the range of 2*θ* = 20–80°. AES was performed using a PHI-700 system in an ultrahigh vacuum (UHV) system under a base pressure below 3.9 × 10^−9^ Torr.

## Supplementary information

Supplementary Information

## Data Availability

The data that support the findings of this study are available from the corresponding author upon request.
