# Supplementary material for: Ultrathin high-κ antimony oxide single crystals
Source: Nat Commun. 2020 May 19;11:2502. doi: 10.1038/s41467-020-16364-9 (PMC7237679; doi:10.1038/s41467-020-16364-9)
Supplement: Supplementary file 1 — Supplementary Information [file 41467_2020_16364_MOESM1_ESM.pdf]

Supplementary Information

Ultrathin high-κ antimony oxide single crystals

Yang et al

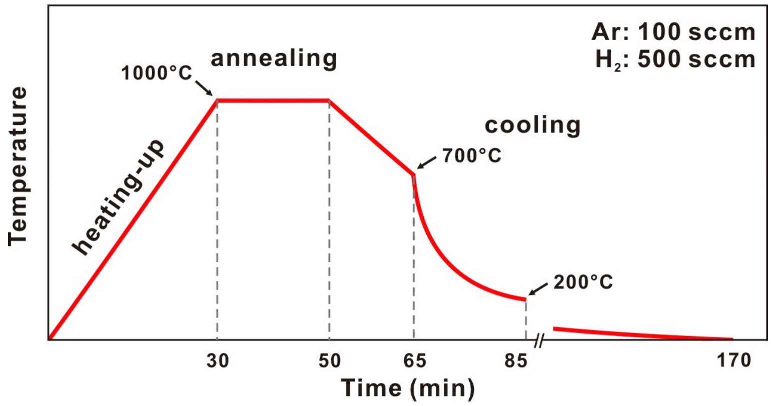

Supplementary Figure 1: Diagram of the annealing procedure utilized for the preparation of re-solidified Ag substrate. For the preparation of re-solidified Ag substrate, a piece of Ag wire was placed on the Co foil. Under the protection of 100 sccm Ar and 500 sccm H_2 atmosphere, Ag melted and then spread evenly over the entire foil by annealing at 1,000ºC for 20 min.

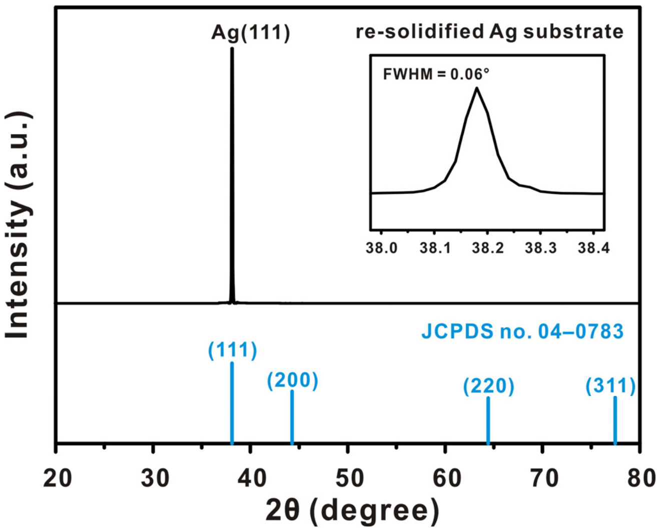

Supplementary Figure 2: X-ray diffraction (XRD) pattern of the re-solidified Ag substrate. Inset, zoom-in image of the (111) peak. After the annealing process shown in Supplementary Fig. 1, XRD investigations of the re-solidified Ag substrate were conducted. The data confirmed that the Ag substrate is crystalline. When referenced with JCPDS data (JCPDS no. 04–0783), a clear match for Ag(111) is obtained. The corresponding zoom-in image of the (111) peak is provided in the inset, which demonstrates that the full width at half maximum (FWHM) of the peak is 0.06°, confirming the high crystallinity of the Ag(111) substrate. Moreover, this was found for the entire surface, which confirms the single crystalline nature of the Ag substrates.

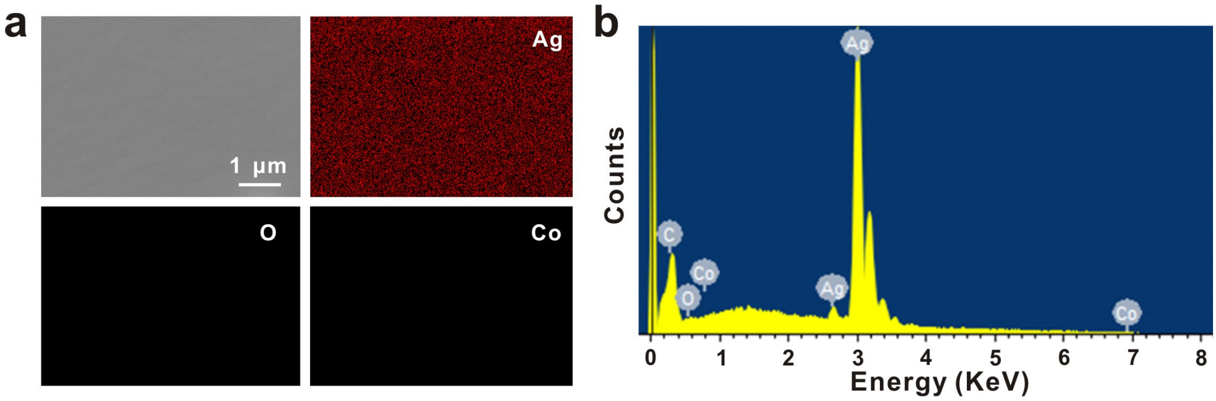

Supplementary Figure 3: Surface elemental composition of the Ag substrate. (a) SEM image and EDS mapping result of the Ag surface. (b) Corresponding EDS spectrum collected on the whole region shown in a. The EDS mapping result confirms that the element of Ag is uniformly distributed on the surface and there is no Co or O element on the substrate. Moreover, as shown in the corresponding EDS spectrum, there is no peak appearing in the regions of Co (K_α: 6.931 keV, L_α: 0.775 keV) and O (K_α: 0.523 keV), which excludes the formation of Ag-Co alloys as well as the oxidation of Ag on the surface.

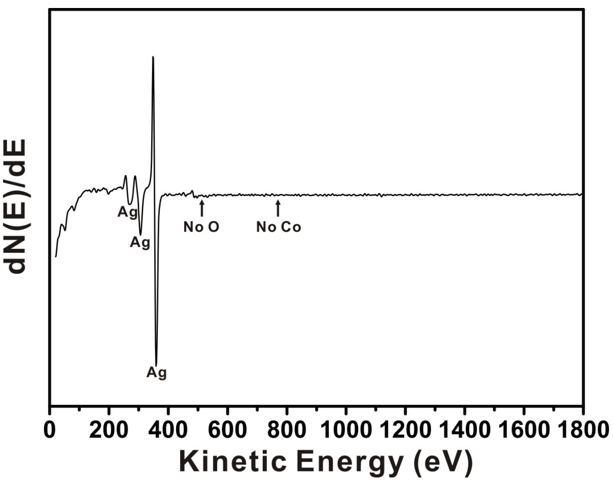

Supplementary Figure 4: Auger electron spectroscopy (AES) result of the Ag substrate. Only Ag peaks appear and there is no peak corresponding to Co and O in the spectrum, which further excludes the formation of Ag-Co alloys as well as the oxidation of Ag on the surface.

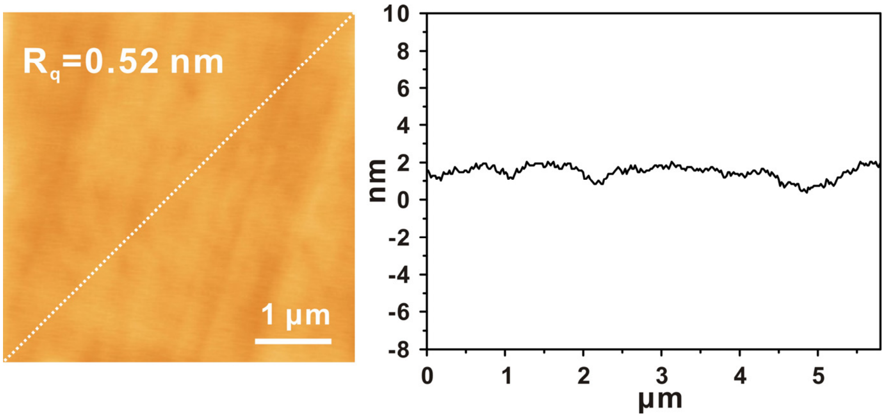

Supplementary Figure 5: Morphology of the surface of the as-prepared Ag substrate, which demonstrates that the root-mean-square roughness (R_q) of Ag surface is 0.52 nm.

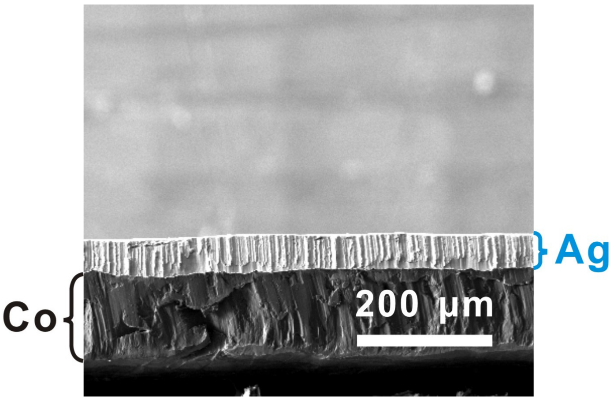

Supplementary Figure 6: Cross-sectional SEM image of the Ag/Co substrate, which demonstrate that the thickness of Ag layer is ~50 μm.

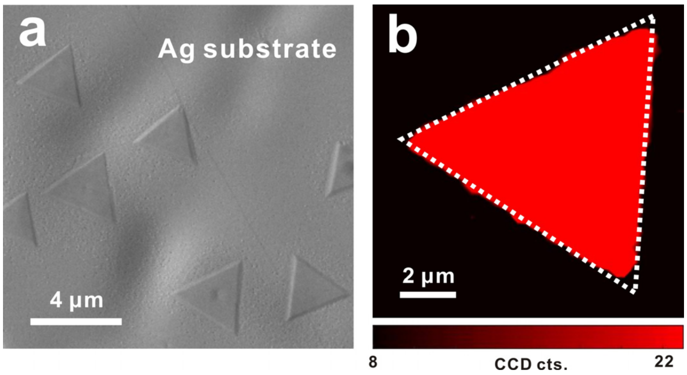

Supplementary Figure 7: The SEM image and Raman mapping result of typical ultrathin antimony oxide crystals. (a) The SEM image of the ultrathin antimony oxide crystals on Ag substrate. (b) Raman mapping result of the A_{1g} peak of an individual ultrathin antimony oxide crystal.

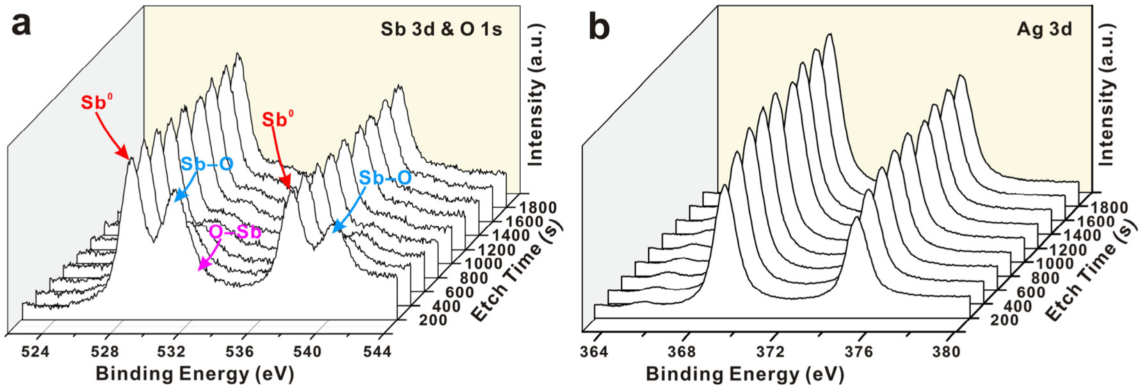

Supplementary Figure 8: X-ray photoelectron spectroscopy (XPS) depth profile of the substrate after the growth. (a–b) Changes of Sb 3d & O 1s core-level signal (a) and Ag 3d core-level signal (b) with respect to the etch time on the Ag substrate after the growth.

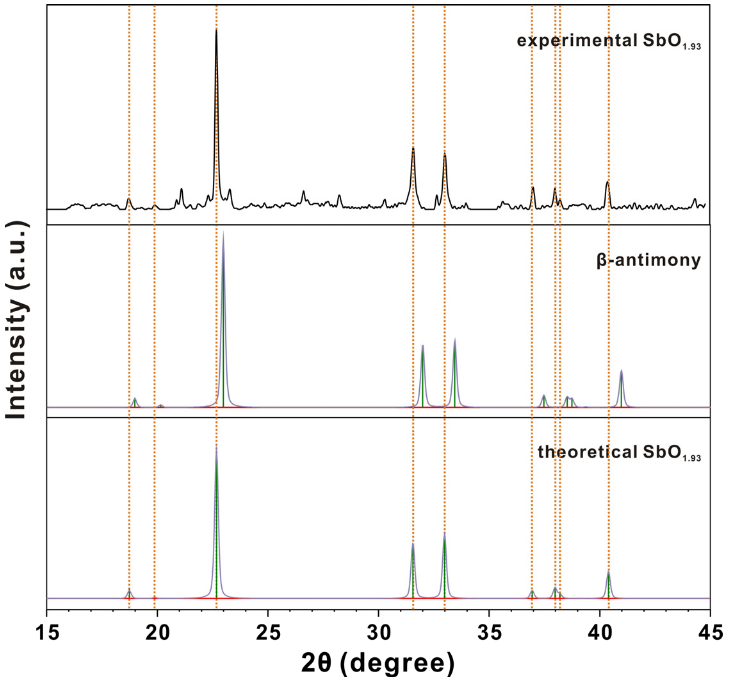

Supplementary Figure 9: The comparison between the XRD pattern of the $SbO_{1.93}$ sample and the simulated XRD patterns of *β*-antimony (Crystallography Open Database (COD) ID: 5000214) and **theoretical $SbO_{1.93}$ structure.**

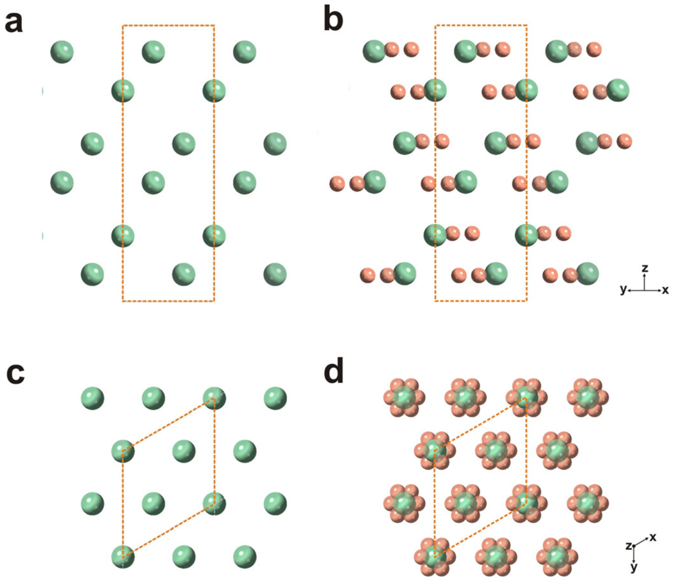

Supplementary Figure 10: The comparison between the structure of *β*-antimony and that of $SbO_{1.93}$. (a–b) the side views of *β*-antimony (a) and $SbO_{1.93}$ (b) (along [100] direction). (c–d) the vertical views of *β*- antimony (c) and $SbO_{1.93}$ (d) (along [001] direction). The black lines are the cell edges. For the *β*-antimony (COD ID: 5000214), the space group is R−3m, the cell parameters are a = b = 4.3084 Å, c = 11.2740 Å.

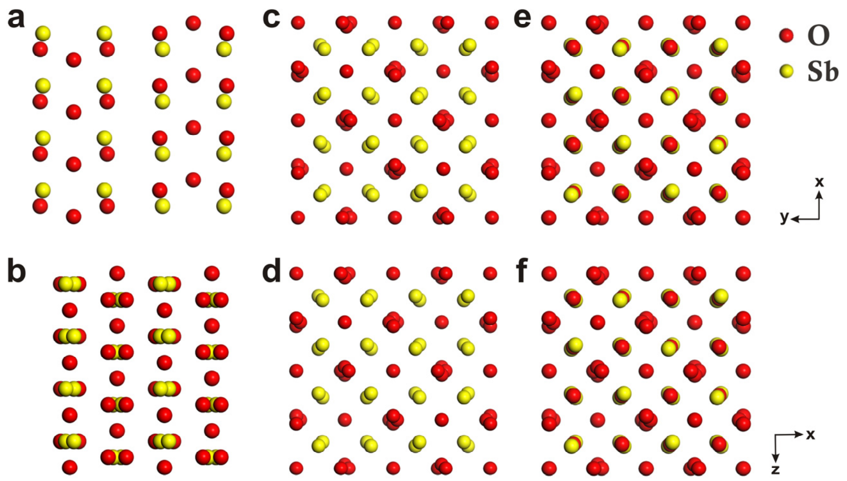

Supplementary Figure 11: Schematic of the structures of other common antimony oxides. (a, b) Top view (a) and side view (b) of monoclinic Sb_2O_3[6]. (c, d) Top view (c) and side view (d) of cubic Sb_2O_3[7]. (e, f) Top view (e) and side view (f) of cubic Sb_2O_5[7].

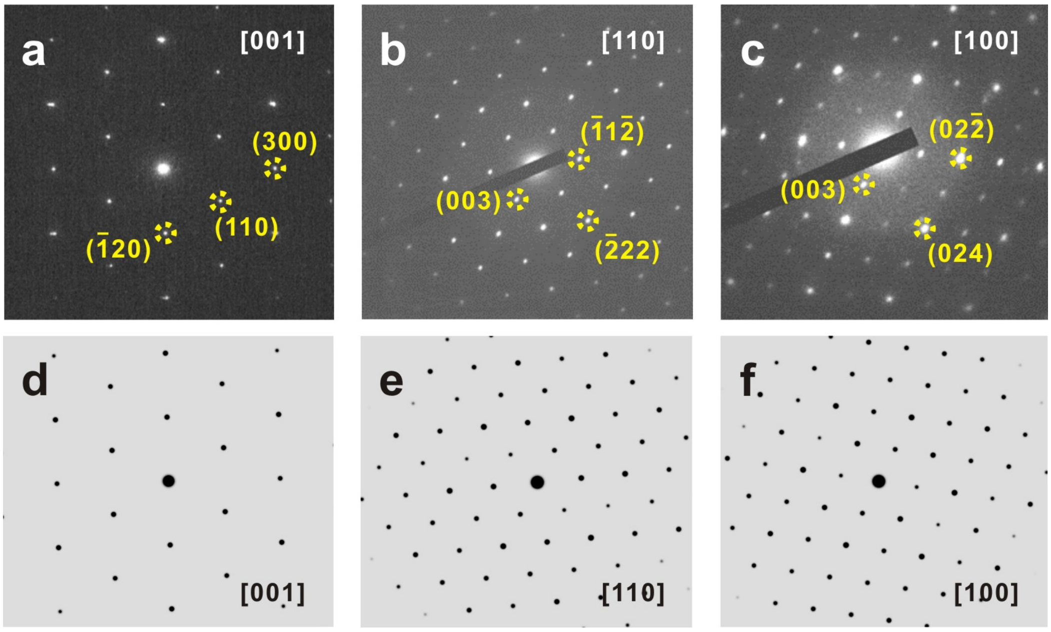

Supplementary Figure 12: The comparison between experimental and simulated selected area electron diffraction (SAED) patterns. (a–c) Representative SAED patterns of the $SbO_{1.93}$ sample along the direction of [001] (a), [110] (b) and [100] (c). (d–f) Simulated SAED patterns of $SbO_{1.93}$ along the direction of [001] (d), [110] (e) and [100] (f).

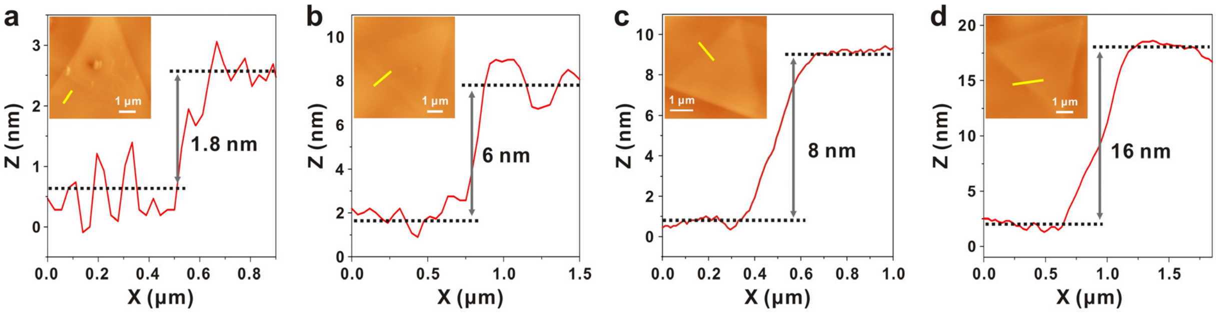

Supplementary Figure 13: Morphology of ultrathin $SbO_{1.93}$ crystals with different thickness. (a−d) Atomic force microscopy (AFM) images of the ultrathin $SbO_{1.93}$ crystals corresponding to 1.8 nm (a), 6 nm (b), 8 nm (c) and 16 nm (d), respectively.

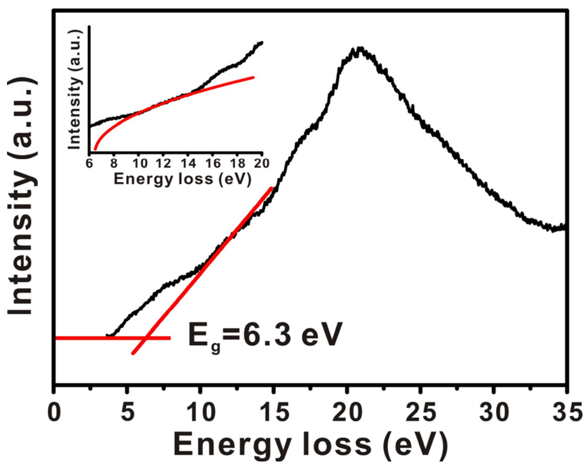

Supplementary Figure 14: The band gap measurement of the ultrathin $SbO_{1.93}$. Plot of the low-loss electron energy loss spectrum (EELS), which provides an estimate of the band gap via linear fit and parabolic fit (inset).

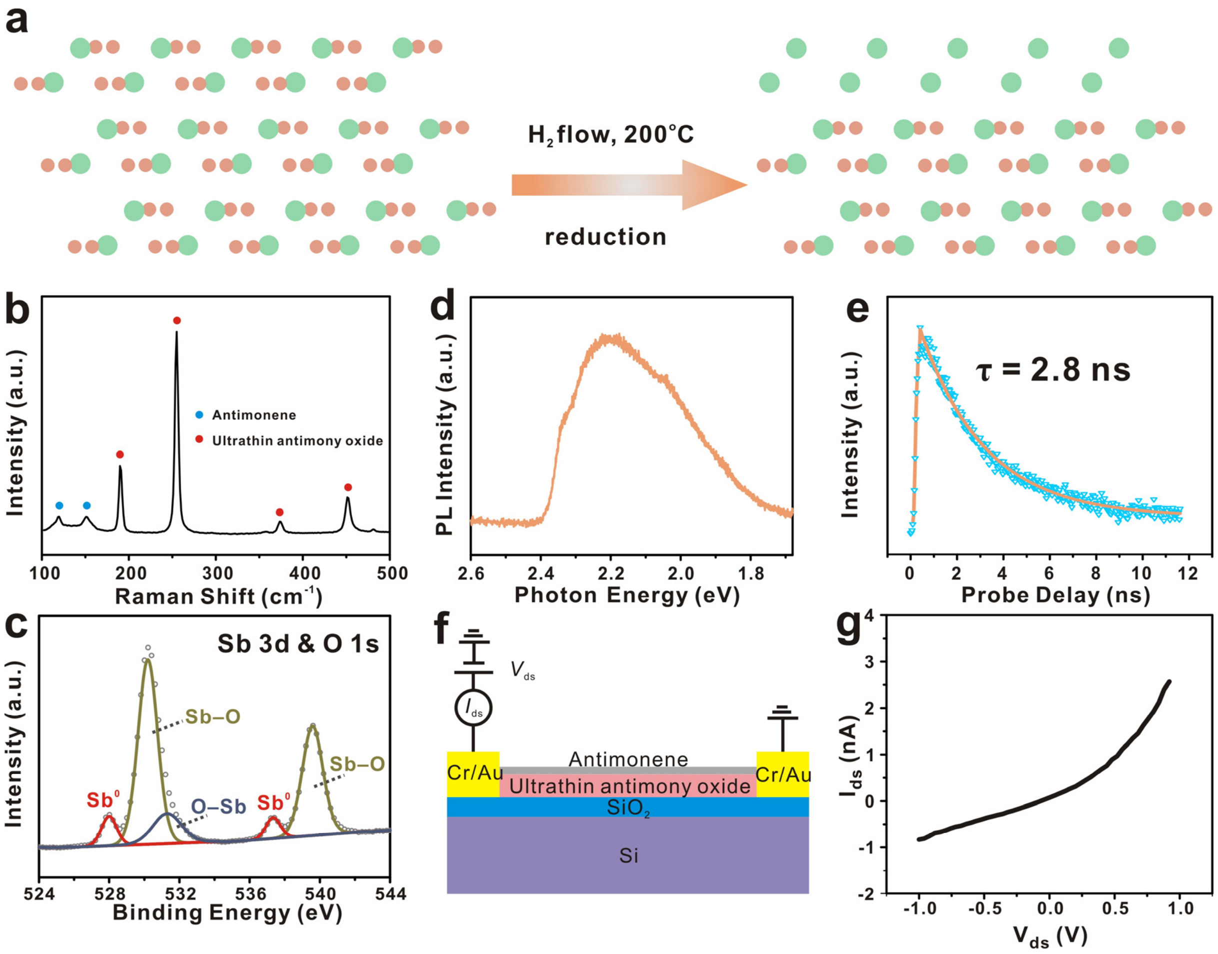

Supplementary Figure 15: The reduction of the ultrathin antimony oxide to antimonene/antimony oxide. (a) Schematic for the reduction process of ultrathin antimony oxide to antimonene/antimony oxide. (b) The Raman spectrum of the antimonene/antimony oxide. (c) The XPS spectra of the antimonene/antimony oxide. (d) The photoluminescence (PL) spectrum of the antimonene/antimony oxide. (e) Time-resolved photoluminescence (TRPL) and fits for the decay time (τ) in the antimonene/antimony oxide. (f) Schematic illustration of the antimonene/antimony oxide device. (g) I_{ds}–V_{ds} characteristics of the tested device in (f).

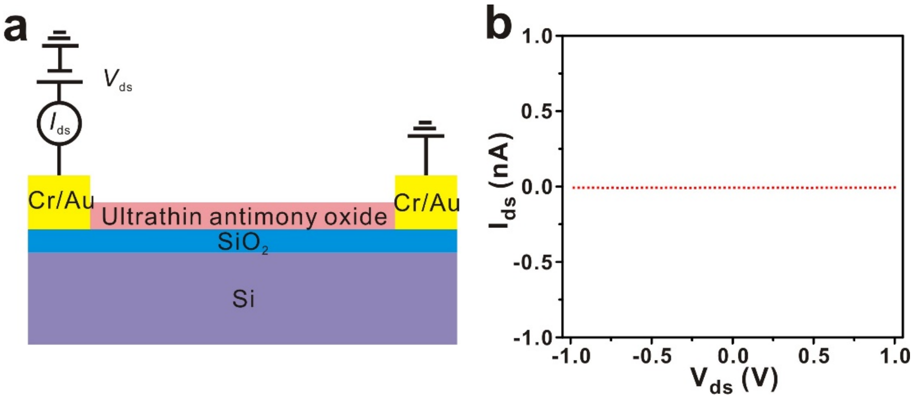

Supplementary Figure 16: The electrical characterization of the ultrathin antimony oxide which shows its excellent insulating property. (a) Schematic illustration of the tested device. (b) I–V characteristics.

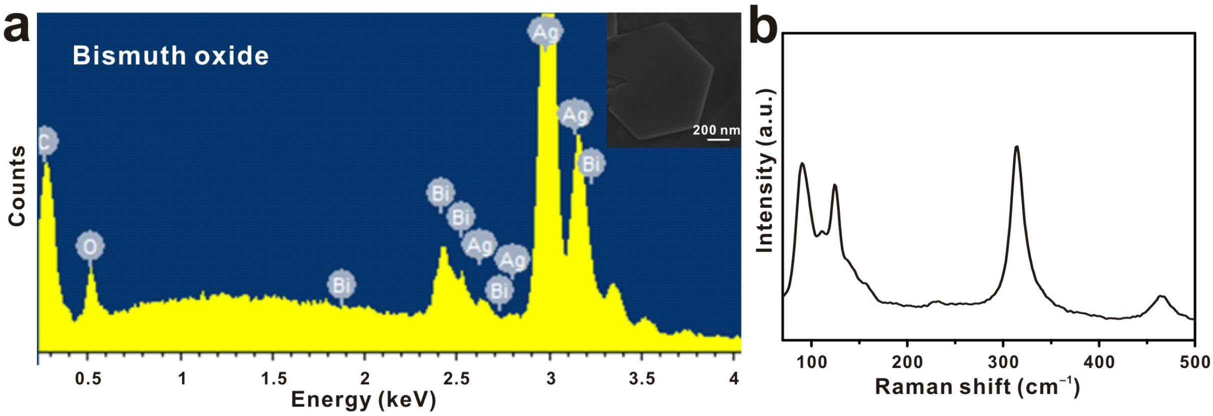

Supplementary Figure 17: Characterizations of the bismuth oxide. (a) SEM image and EDS element analysis result of the as-grown bismuth oxide. (b) Raman spectrum of bismuth oxide.

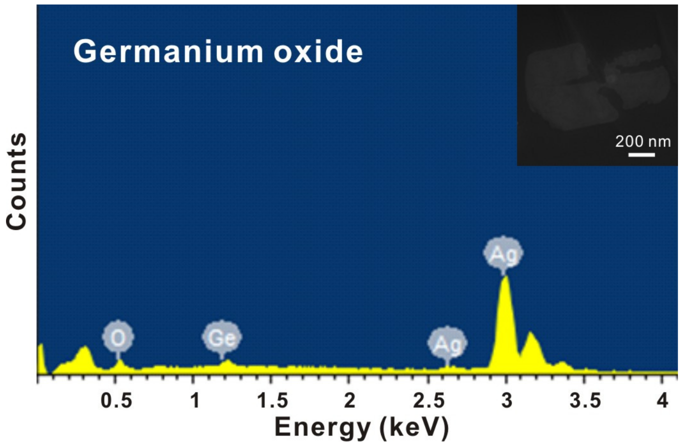

Supplementary Figure 18: SEM image and EDS element analysis result of the as-grown germanium oxide.

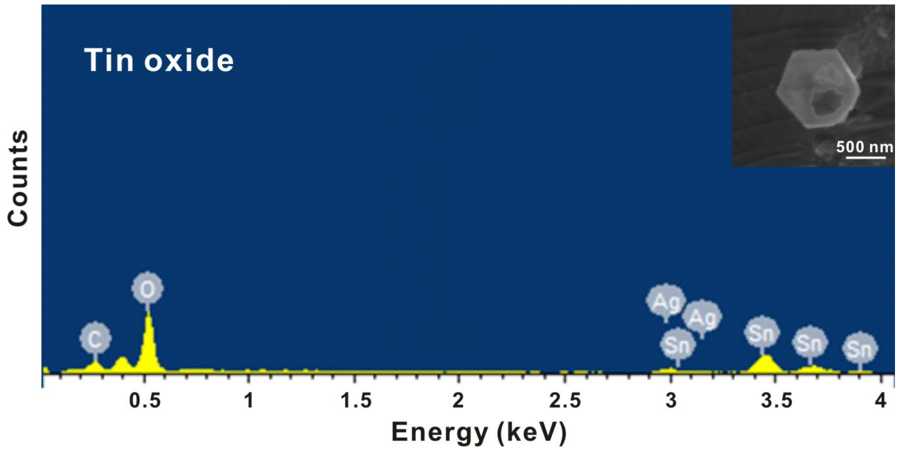

Supplementary Figure 19: SEM image and EDS element analysis result of the as-grown tin oxide.

Supplementary Table 1: Crystal data and structure refinement for $SbO_{1.93}$.

**Empirical formula**	$SbO_{1.93}$
**Formula weight**	152.64
**Temperature**	298 K
**Wavelength**	1.2398 Å
**Crystal system**	Trigonal
**Space group**	*R* −3/*m (166)*
**Unit cell dimensions**	$a = b = 4.3658(8)$ Å; $c = 11.4256(23)$ Å<br>$\alpha = \beta = 90°$; $\gamma = 120°$
**Volume**	188.60(9) Å³
**Z**	6
**Density (calculated)**	12.3564 g/cm³
$R_{\omega p}$	0.3917
R_p	0.3488
χ^2	1.605
**θ range for data collection**	15 to 45°
**Data / restraints / parameters**	14
**Goodness-of-fit**	1.27

Supplementary Table 2: Wyckoff sites, atomic coordinates, occupancy factors (f_{occ}) and isotropic displacement parameters (U_{iso}) for $SbO_{1.93}$.

Label	Wyckoff sites	*x*	*y*	*z*	f_{occ}	U_{iso} / Å^2
**Sb**	6*c*	0.6667	0.3333	0.0967(5)	1.00	0.055(6)
**O**	18*g*	0.188(2)	0.594(9)	0.243(3)	0.64(9)	0.2500

Supplementary Table 3: Bond lengths (Å) and bond angles (°) for $SbO_{1.93}$ with estimated standard deviations in parentheses.

Bond names	Bond lengths / Å	Bond angle names	Bond angles / °
**Sb-O × 3**	2.59(6)	**Sb-O-Sb × 2**	96.2(8)
**Sb-O × 3**	2.20(4)	**Sb-O-Sb × 1**	143.7(3)
**Sb-O × 6**	2.30(2)		

Supplementary Table 4: The extracted diffraction data by Rietveld refinement[8].

2θ	(hkl)	d (Å)	Intensity (f)
18.738	$(00\bar{3})$	3.8085	5.4
19.893	$(\bar{1}11)$	3.5895	0.8
22.681	(012)	3.1529	100
31.571	$(\bar{1}14)$	2.2791	37.6
33.001	$(\bar{1}20)$	2.1829	43.6
36.966	(015)	1.9557	5.5
38.002	$(00\bar{6})$	1.9043	7.3
38.219	$(\bar{1}23)$	1.8939	3.0
38.832	(021)	1.8651	0.1
40.419	$(\bar{2}22)$	1.7947	18.4

Supplementary Table 5: Growth parameters for various ultrathin oxides.

	Growth Temperature	Precursor	Carrier Gas
Antimony Oxide	750°C	Sb powder	20 sccm Ar<br>5 sccm H_2 and O_2
Bismuth Oxide	850°C	Bi powder	20 sccm Ar<br>5 sccm H_2 and O_2
Germanium Oxide	930°C	Ge powder	14 sccm Ar<br>3 sccm H_2 and O_2
Tin Oxide	930°C	Sn powder	8 sccm Ar<br>3 sccm H_2 and O_2

Supplementary Note 1: Structural analysis process.

The starting structure can be obtained by the structure determination from the experimental data of GI-XRD. First, all the major diffraction peaks are well indexed to fit the hexagonal symmetry using the software MDI Jade 6.0 (Materials Data, Inc.) and the possible space group is proposed as R−3m by the software CheckCell. After that, the diffraction intensities are successfully extracted and transferred into a "*.hkl" file by the software PowderCell. Then, both the "*.ins" file from *β*-antimony and the as-prepared "*.hkl" file of ultrathin antimony oxide are read by the software SHELX and refined until the goodness of fit is reduced below the acceptable limit. During this stage, the atomic positions of Sb and O are both confirmed by the Pattson method. Finally, a modified "*.cif" file for *β*-antimony is produced which can be recognized as the starting structure model for further structural refinement (Rietveld refinement) by the software GSAS-EXPGUI.

Supplementary Note 2: A discussion of the obtained $SbO_{1.93}$ structure compared to other possibilities.

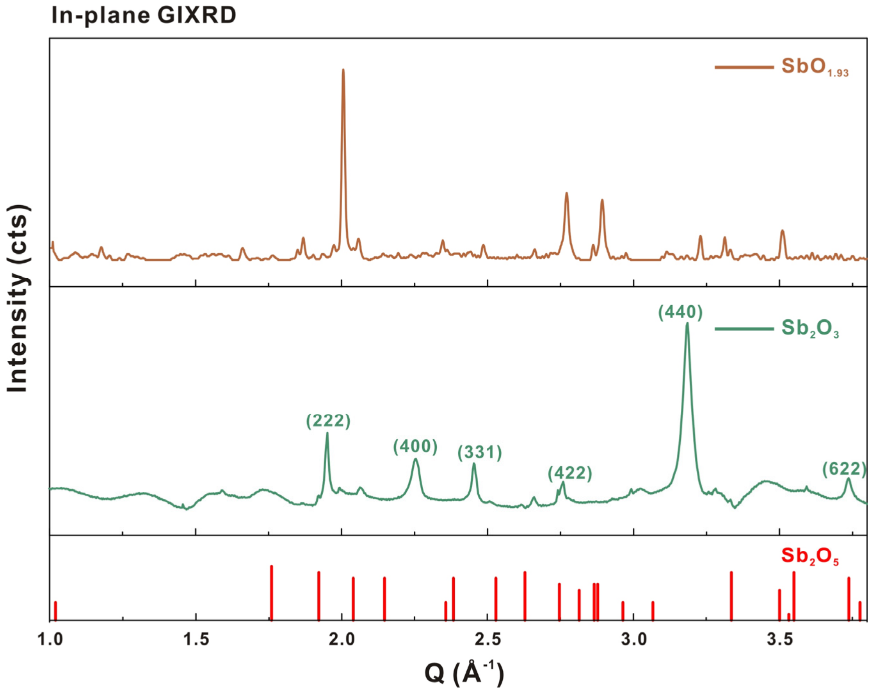

Supplementary Figure 20: The comparison diagram of XRD spectra. The comparison diagram of synchrotron-based GI-XRD spectra between $SbO_{1.93}$ and Sb_2O_3. The diffraction data of Sb_2O_5 (Joint Committee on Powder Diffraction Standards (JCPDS) 34-0878) are also provided for reference.

Supplementary Fig. 20 presents that the diffraction patterns of the ultrathin antimony oxide ($SbO_{1.93}$) are quite different from those of Sb_2O_3 (JCPDS no. 05-0534) and Sb_2O_5 (JCPDS no. 34-0878), which confirms that the ultrathin antimony oxide is not a mixture of Sb_2O_3 and Sb_2O_5. Besides, the obtained structure and stoichiometry are constrained sufficiently under the current experimental conditions, and there is still room for the improvement of precision of oxygen content with the improvement of experimental conditions.

Supplementary Note 3: Schottky emission model

The fitting of the data is described as follows:

Current voltage curves (I–V) were obtained during AFM-based peak force tunneling characterization. The observed I–V characteristics follow the Schottky emission model, which can be described as[9,10]:

$$J = \frac{4\pi qK^2m^*}{h^3}T^2e^{\frac{-q(\phi_B-\sqrt{qE/4\pi\varepsilon_r\varepsilon_0})}{kT}} \tag{1}$$

in which, J is the current density, q is the elementary charge of an electron, K is Boltzmann's constant, $m*$ is the effective electron mass in the dielectric, h is Planck's constant, T is the temperature in Kelvin, $\varnothing_B$ is the Schottky barrier height, E is the electric field, ε_0 is the permittivity of vacuum and ε_r is the relative dielectric constant. The current density can be derived as follows:

$$J = \frac{I}{A^*} \tag{2}$$

In which, I is the current and $A*$ is the effective AFM tip contact area. We have rewritten Equation (1) as seen below:

$$I = A^*\frac{4\pi qK^2m^*}{h^3}T^2e^{\frac{-q(\phi_B-\sqrt{qE/4\pi\varepsilon_r\varepsilon_0})}{kT}} \tag{3}$$

To estimate the dielectric constant, we have further simplified the equation:

$$I = \alpha e^{\frac{-q(\phi_B-\sqrt{qE/4\pi\varepsilon_r\varepsilon_0})}{kT}} \tag{4}$$

Where α is defined as follows:

$$\alpha = A^*\frac{4\pi qK^2m^*}{h^3}T^2 \tag{5}$$

The logarithm of Equation (4) gives:

$$Ln(I) = Ln(\alpha) + \frac{-q(\phi_B-\sqrt{qE/4\pi\varepsilon_r\varepsilon_o})}{kT} \tag{6}$$

The electric field across the dielectric film is defined as:

$$E = \frac{V}{d} \tag{7}$$

where V is the applied voltage and d is the dielectric thickness. The thickness was measured to be ~1.5 nm using AFM.

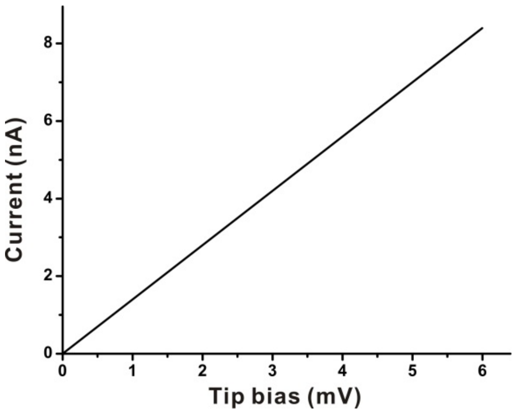

Supplementary Figure 21: Conductive AFM test for Ag substrate. I–V curve using the peak force tunneling AFM (PF-TUNA) experimental setup shown in Fig. 4a in the main text, measuring the I–V curve of the conductive tip when in direct contact with the Ag substrate.

Supplementary Fig. 21 shows the I–V curve of the conductive tip directly placed on the Ag substrate (without antimony oxide), which indicates that the resistance of the experimental setup is negligible and that the measured I–V characteristics (Fig. 4d in the main text) is entirely associated with the ultrathin antimony oxide. Fitting of the experimental data (Fig. 4d in the main text) to Equation (6) was conducted and the estimated relative dielectric constant is ~100 with $R^2 = 0.86$. The fit is shown in the inset of Fig. 4d in the main text.

Supplementary Note 4: Bandgap determination using electron energy loss spectroscopy (EELS)

The low-loss section of the EELS spectra is corresponding to the zero loss peak that has been attributed to the probe beam. For wide bandgap semiconductors and dielectrics, the zero loss peak is followed by a flat region (~zero intensity) that is again followed by a sharp rise in intensity at the material's bandgap energy. As a result, low loss EELS analysis can be utilized to measure the bandgap of materials. According to the reference[11], we applied the linear fit method to determine the band gap. Analysis of the EELS spectrum shown in Supplementary Fig. 14 results in an estimated bandgap of E_g = 6.3 eV. An alternative approximation is based on another previous work reported by Rafferty and Brown[12], which considers a mathematical fit of the form: Intensity = $\alpha(E - E_g)^b$, where α is scaling parameter, E is the energy, E_g is the band gap energy, and b takes the value of 0.5 or 1.5 depending whether it is direct transition or indirect transition. An estimate of the band gap using the fit of $\alpha(E - E_g)^{0.5}$ suggests a direct electronic transition. An excellent fit is observed with $R^2 = 0.98$. This fit is shown in the inset of Supplementary Fig. 14, and this parabolic fit indicates that the bandgap is also measured to be 6.3 eV.

Supplementary References

1. Perdew, J., Burke, K., Ernzerhof, M. Generalized gradient approximation made simple. *Phys. Rev. Lett.* **77**, 3865−3868 (1996).

2. Blöchl, P. E. Projector augmented-wave method. *Phys. Rev. B* **50**, 17953−17979 (1994).

3. Mak, K. F., Lee, C., Hone, J., Shan, J., Heinz, T. F. Atomically thin MoS_2: A new direct-gap semiconductor. *Phys. Rev. Lett.* **105**, 136805 (2010).

4. Togo, A., Oba, F., Tanaka, I. First-principles calculations of the ferroelastic transition between rutile-type and $CaCl_2$ type SiO_2 at high pressures. *Phys. Rev. B* **78**, 134106 (2008).

5. Setyawan, W., Curtarolo, S. High-throughput electronic band structure calculations: Challenges and tools. *Comp. Mater. Sci.* **49**, 299−312 (2010).

6. Buerger, M. J. *American Mineralogist* **21**, 206−207 (1936).

7. Dehlinger, U. *Zeitschrift fur Kristallographie* **66**, 108−119 (1927).

8. Peterson, V. K. Lattice parameter measurement using Le Bail versus structural (Rietveld) refinement: A caution for complex, low symmetry systems. *Powder Diffr.* **20**, 14−17 (2005).

9. Jahanmir, J., West, P. Evidence of Schottky emission in scanning tunneling microscopes operated in ambient air. *Appl. Phys. Lett.* **52**, 2086−2088 (1988).

10. Chiu, F. C. A review on conduction mechanisms in dielectric films. *Adv. Mater. Sci. Eng.* **2014**, 18 (2014).

11. Damasio, M.-C., Francisco, P.-D., Raúl, B.-U., Sofía, B.-C., Guillermo, H.-P., Paolo, L., Marek, M. Band gap measurement of $Bi_2Mo_xW_{1-x}O_6$ by low loss electron energy loss spectroscopy. *Mater. Sci. Semicon. Proc.* **63**, 184−189 (2017).

12. Rafferty, B., Brown, L.M. Direct and indirect transitions in the region of the band gap using electron-energy-loss spectroscopy. *Phys. Rev. B.* **58**, 10326−10337 (1998).
